# Dentin Morphology of Root Canal Surface: A Quantitative Evaluation Based on a Scanning Electronic Microscopy Study

**DOI:** 10.1155/2015/164065

**Published:** 2015-08-27

**Authors:** Giuseppe Lo Giudice, Giuseppina Cutroneo, Antonio Centofanti, Alessandro Artemisia, Ennio Bramanti, Angela Militi, Giuseppina Rizzo, Angelo Favaloro, Alessia Irrera, Roberto Lo Giudice, Marco Cicciù

**Affiliations:** ^1^Department of Medical-Surgery and Odontostomatologic Experimental Sciences, University of Messina, Italy; ^2^Department of Biomedical Sciences and Morpho-Functional Imaging, University of Messina, Italy; ^3^IRCCS Centro Neurolesi “Bonino-Pulejo”, Messina, Italy; ^4^IPCF-CNR Viale Stagno D'Alcontres, 98100 Messina, Italy; ^5^Department of Human Pathology, University of Messina, Via Consolare Valeria, 98100 Messina, Italy

## Abstract

Dentin is a vital, hydrated composite tissue with structural components and properties that vary in the different topographic portions of the teeth. These variations have a significant implication for biomechanical teeth properties and for the adhesive systems utilized in conservative dentistry. The aim of this study is to analyse the root canal dentin going from coronal to apical zone to find the ratio between the intertubular dentin area and the surface occupied by dentin tubules varies. Observations were conducted on 30 healthy premolar teeth extracted for orthodontic reasons in patients aged between 10 and 14. A SEM analysis of the data obtained in different canal portions showed that, in the coronal zone, dentinal tubules had a greater diameter (4.32 *μ*m) than the middle zone (3.74 *μ*m) and the apical zone (1.73 *μ*m). The average number of dentinal tubules (in an area of 1 mm^2^) was similar in coronal zone (46,798 ± 10,644) and apical zone (45,192 ± 10,888), while in the middle zone they were lower in number (30,940 ± 7,651). However, intertubular dentin area was bigger going from apical to coronal portion. The differences between the analysed areas must be considered for the choice of the adhesive system.

## 1. Introduction

Dentin is the calcified tissue that forms the major part of the tooth. It is composed mainly by type I collagen fibrils (and a small amount of types III, IV collagen, noncollagen proteins, and proteoglycans) and by hydroxylapatite [1–3].

Anatomic dentin microstructure shows dentinal tubules, cylindrical canals of 1-2 *μ* in diameter, running from the pulp to the dentinoenamel junction (DEJ) in the crown, and the cementodentinal junction (CEJ) in the root. An intertubular dentin layer individually surrounds these tubules. Calcified collagen fibrils are 50–100 *μ* of average diameter and constitute the dentin basic structure; they are orthogonal to the tubules and form an intertubular dentin matrix network [1, 4].

In order to evaluate dentin ultrastructure several studies [5, 6] and many different techniques have been performed immunofluorescence, microradiography, scanning electron microscopy (SEM), and transmission electron microscopy (TEM).

A detailed knowledge of dentin structure is essential in order to understand its physiology and the mechanism by which different adhesive systems work in restorative dentistry. In the total etching technique we use the exposed intertubular collagen fibrils together with resin tubular tags to obtain a >20 MPa adhesion force [5–8].

The majority of teeth anatomical studies analyse the coronal dentinal substratum. Cagidiaco and Ferrari [6] demonstrated how the anatomy of the coronal dentin is characterized by different density, diameter, and orientation of dentinal tubules in different cavity preparation cutting planes.

From the literature analysis, observing the coronal part of the dentin layer close to the pulp, the dentinal tubules number was 65,000–45,000/mm^2^; this number was higher if compared to the outer dentin areas (15,000–20,000/mm^2^) [9, 10].

The tubular diameter is larger near to the pulp (3-4 *μ*) and smaller in the peripheral area near to the DEJ (average diameter 1.7 *μ*). Casually large dentinal tubules have been observed [11].

Age determines variations of the tubular lumen diameter due to a physiological sclerosis of the dentinal tubules; indeed, in advanced age, the tubules located in the most superficial dentin layer may measure even 0.2 *μ* [12].

Dentinal tubules have very thin collateral ramifications (1 *μ* diameter). These secondary tubules are right-angled, divided, and connected to closer tubules through intertubular dentin forming a three-dimensional network [11, 13].

We can state that tubule morphology and intertubular substance differences were found in coronal and root dentin as well as a wide variation among different areas of the root canal [14, 15].

This micromorphological study is aimed to evaluate, in vitro, dentinal tubules size and tubular distribution in coronal, middle, and apical root portions. This anatomical condition, related to the intertubular dentin area and the surface occupied by dentinal tubules (determined by their number and diameter), may influence the adhesives efficiency in endocanal cementation of composite reinforced posts.

## 2. Materials and Methods

Observations were conducted on 30 healthy premolars extracted for orthodontic reasons, in patients aged between 10 and 14 (mean age: 11.4 years, STD 1.26) and preserved in saline solution (0.9%) at 4°C. Informed consent was obtained from all patients and all the procedures were performed according to the Helsinki Declaration of 1975.

The preparation procedure of specimens consisted in a preliminary tooth crown and a root pulp tissue removal.

Pulp removal was performed using manual endodontic files under irrigation of 5% NaOCl (Niclor 5; OGNA Lab S.r.l Muggiò, MB, IT) at 50°C, alternating with 17% EDTA (OGNA Lab S.r.l Muggiò, MB, IT) for 20 min followed by a wash with 5% NaOCl for 1 min and a saline solution (OGNA Lab S.r.l Muggiò, MB, IT).

Preliminary to the observation we proceeded by etching the canal lumen with 37% orthophosphoric acid (Universal Etchant Scotchbond; 3M ESPE, St. Paul, USA) for 15 sec., washing with saline solution and metallizing [16]. Afterwards, all specimens were divided along the longitudinal axis using a coronal-apical groove such as a fracture guide.

All specimens were analysed with Gemini Field Emission SEM (FE-SEM) SUPRA 25 (Carl Zeiss NTS GmbH, Oberkochen, Germany), with an EDAX EDX detector. Surface was analysed with a 1.7 *μ*m resolution (15 kV) at a 3072 × 2304 pixels resolution. A 2,500x was used to quantify dentinal tubules density and intertubular surface, while a 23,000x was used to evaluate tubule morphology. Three different areas (coronal, middle, and apical) of the root canal of each specimen were examined [16, 17]. Within the same area of the canal, measurement was conducted by taking random references from three default areas that were 400 *μ*m^2^. According to anatomical observations, the number of tubules in the three measurement areas was quantified and the tubules average number was calculated in each area (Table 1). On 9 tubules randomly selected within each area (3 for each area) the diameter was measured (Table 1).

According to these data, quantification of the dentinal surface area occupied by the tubular lumens (in absolute value and percentage) and intertubular dentin surface area was made.

The aforementioned quantification was carried out as follows.

(1) Identification of the number of dentinal tubules per mm^2^: this parameter was calculated by the Schellenberg formula (*X* = *n*10^6^/*z*), where *n* is the number of tubules observed in each analysed area and *z* is the global surface of observation (Table 2).
(2) Identification of tubular lumen average surface: considering the tubule section is roughly circular, and the area was obtained through its average diameter (Table 2).
(3) Calculation of area occupied by all tubules: this datum was obtained by multiplying tubules number in one mm^2^ to tubules average area (Table 2).
(4) The intertubular dentin surface area was obtained by subtraction of the area occupied by the tubules (Table 2).

A further datum was obtained by calculating the percentage ratio between the observation area and the tubular lumen area (Table 2).

A two-way ANOVA test is a way for investigating the effect of two nominal predictor variables on a continuous outcome variable. For this reason two-way analysis of variance (ANOVA) was performed to verify statistically significant difference among the tested groups. A *P* value < 0.05 was considered as significant. Then we considered the post hoc doc accordingly with LSD Fisher' test: $\text{LSD}=t_{\alpha /2,df}\sqrt{2S_{e}^{2}/n}$ in order to have the significance values [18].

## 3. Results

**Figure 1:**
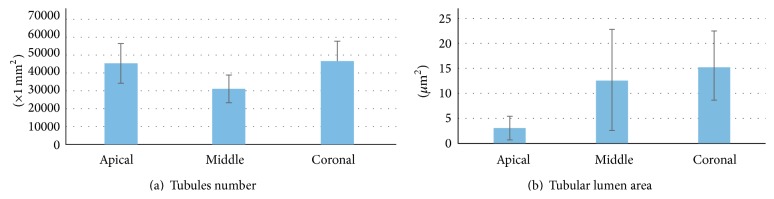
Dentinal tubules number (in 1 mm^2^) (a) and tubular lumen area (*μ*m^2^) (b) in each root portion.

The analysis of the data obtained from the microphotographs taken in various canal portions is summarised in Tables 1 and 2 and Graphs (Figures 1(a) and 1(b)).

Table 1 shows averages, found in each specimen, and standard deviation of the number of tubules identified in the three random areas, carried out in the coronal, middle, and apical root canal portions.

**Figure 2:**
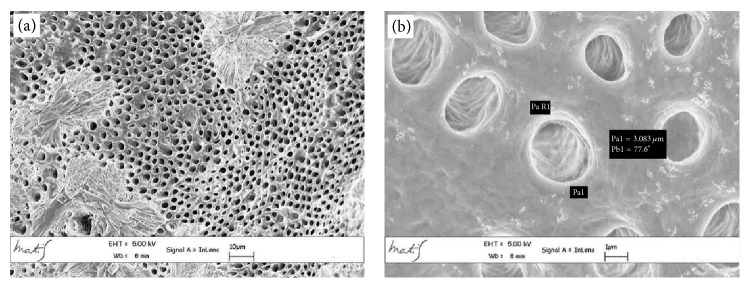
Coronal area metallised dentin specimens analysed with Gemini Field Emission SEM (FEM-SEM) at 1.7 *μ*m and 3072 × 2304 pixel resolution 2500x (a) and 23000x (b) magnification: presence of calcospherites (Figure 3(a)).

**Figure 3:**
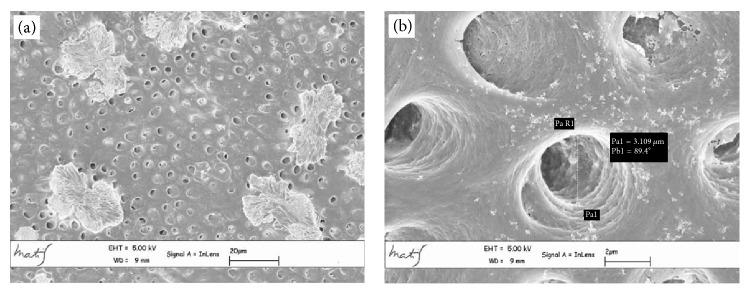
Middle area metallised dentin specimens analysed with Gemini Field Emission SEM (FEM-SEM) at 1.7 *μ*m and 3072 × 2304 pixel resolution 2500x (a) and 23000x (b) magnification: presence of calcospherites (Figure 4(a)).

**Figure 4:**
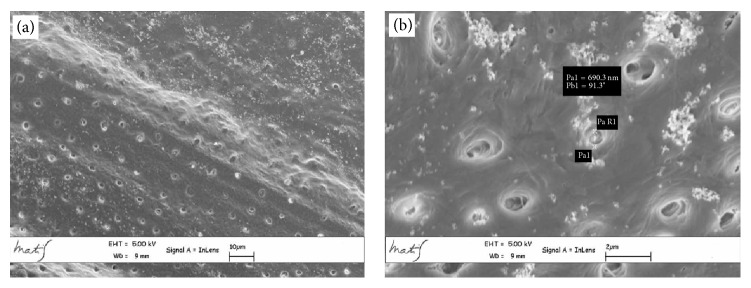
Apical area metallised dentin specimens analysed with Gemini Field Emission SEM (FEM-SEM) at 1.7 *μ*m and 3072 × 2304 pixel resolution 2500x (a) and 23000x (b) magnification.

The average linear values of the dentinal tubules diameters evaluated in 9 areas, expressed in *μ*m, are listed in Table 2. The average diameter within the various areas has a variation range between 6.43 and 2.44 *μ*m (coronal area Figures 2(a) and 2(b)), 6.64 and 1.7 *μ*m (middle area Figures 3(a) and 3(b)), and 3.36 and 0.41 *μ*m (apical area Figures 4(a) and 4(b)).

General averages calculated in mm^2^ show that the coronal third of the canal has a higher tubular density than the middle third (46, 798 ± 10, 644 mm^2^ versus 30, 940 ± 7, 651 mm^2^). In the same areas the tubules average diameter decreases (4.324 *μ*m versus 3.749 *μ*m). The apical third shows an average tubular density of 45, 192 ± 10, 888 mm^2^ that is similar to the coronal third, but with smaller tubular diameter (1.731 *μ*m) (Table 1).

The ANOVA analysis shows that differences between tubular diameter and number observed among the three canal areas (apical versus middle, middle versus coronal) and the ones evaluated by post hoc Fisher's test ($\text{LSD}=t_{\alpha /2,df}\sqrt{2S_{e}^{2}/n}$) result significant (*P* < 0.05).

As shown in Table 2 and Figure 2, the ratio between tubular lumen area and dentinal surface, moving from coronal to apical areas, decreases from 72.42% to 39.53% and 13.71%.

**Figure 5:**
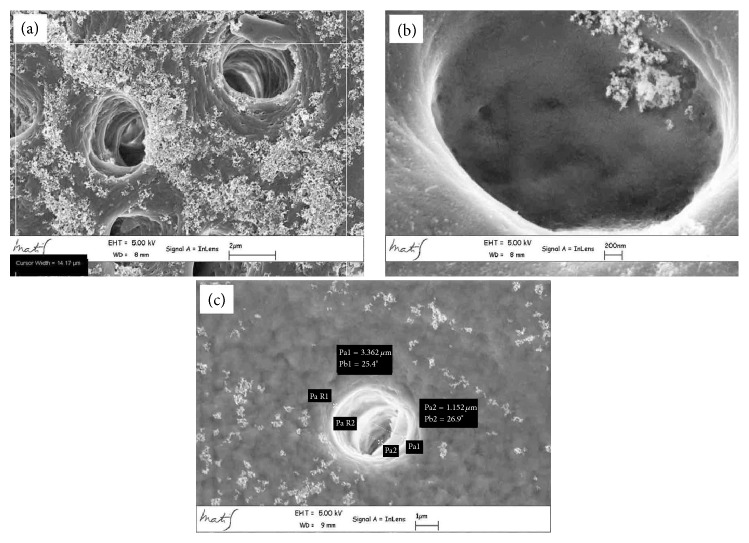
High magnification microphotograph analysis shows a higher number of secondary tubules access cavities in coronal zone than (a) in the middle (b) and apical (c) ones. In Figure (c) the Pa1 is 3.362 *μ*m and it represents the external diameter while the Pa2 is 1.152 *μ*m and it represents the diameter of the internal portion of the tubule.

Furthermore, analysing individually high-magnification microphotographs, the secondary tubules appear to be more common inside the dentinal tubule wall in the coronal portion than those observed in the middle and apical portions (Figures 5(a), 5(b), and 5(c)).

## 4. Discussion

The restoration of teeth treated with endodontic therapy frequently requires the use of endocanalar post cemented with adhesive resin, in order to provide the retention of the coronal restoration and to achieve a better homogeneity between the composite inlay, the build-up, the fiber posts, and the luting agents, reinforcing the residual dental structure [19, 20].

Ferrari et al. [21] report that the anatomical variations that are present between different dentin portions of the root canal can influence the efficiency of the adhesive system used; therefore, the knowledge of details related to canal dentinal structure as well as its tubules and their ramifications is essential to develop efficacious resinous build-ups, adhesives, and endodontic cements [5].

Several authors [21–24] described anatomical variations of both number and size of dentinal tubules, when moving from the coronal to the apical portion of the root canal.

Data retrieved from our research show a substantial morphological variability among the dentin that forms the different endocanalar regions; this variability is seen in tubular number differences and diameter differences. Moving forward from coronal to apical zone our study shows how the tubular lumen area progressively decreases from 15.47 ± 7.06 *μ*m^2^ of the coronal zone to 12.77 ± 10.23 *μ*m^2^ of the middle zone, reaching his minimum of 3.033 ± 2.43 *μ*m^2^ in the apical zone.

Tubular distribution does not seem to be regular moving from 46, 798 ± 10, 644 mm^2^ to 30, 940 ± 7, 651 mm^2^ and to 45, 192 ± 10, 888 mm^2^, respectively, in the coronal, middle, and apical zones.

From the crossed analysis of this data it is evident how the surface occupied by the intertubular dentin, calculated by difference, progressively increases moving from coronal to apical while the ratio between tubular lumen area and dentinal surface progressively decreases (Table 2, Figure 2).

The endocanalar structural differences resulted from our data analysis appear to be substantially influenced by the diameter instead of the tubular number.

Although some authors consider that the endocanalar zone does not affect adhesion, further studies have revealed differences [25, 26].

According to the authors, the push-out test shows higher bond strength values in the apical third than those in other parts of the root canal [27–30].

Differences, in the endocanalar regions, regarding the efficiency of various adhesive systems (total-etch and self-etching adhesive systems) are reported in the literature.

A study by Perdigão et al. [24] showed higher bond strength values in the coronal region using total-etch system. This kind of adhesive exploits both a resin-collagen hybrid layer formation and a micromechanical retention with resin tags inside the dentinal tubules.

The presence of numerous tubules with big diameter and secondary tubule access cavities, identified in our research, can promote this mechanism [31].

Instead in the apical region, the self-etching adhesives that exploit all the dentinal surface show better performances, because the adhesion with these systems is obtained modifying the collagen fibers present in the intertubular dentin area [25, 28, 30].

Our observations agree with this thesis and explain their inner mechanisms, showing the presence of numerous small diameter tubules and a wide intertubular area surface.

Considering this observation, the dentin, as interface of endocanalar adhesion, has to be studied not only considering its tubular number but also considering its diameter and consequently the intertubular dentin characteristics that, biologically, are strictly linked to the odontoblast activities that induce and regulate the mineralization [32].

These cells are involved in type I collagen synthesis and the secretion of proteoglycans and noncollagenous proteins, increasing the level of mineralization of secondary dentin.

The secondary dentin deposition is associated with odontoblasts reorganization in a single layer and determines the decrease in the number of odontoblasts [9].

Bjørndal and Thylstrup demonstrated a low frequency of disjunctions between the odontoblast layer and the predentin in the undermineralized tooth [33].

In presence of carious lesions further mineralization and dentinal anatomy modifications are evident, along with the tertiary dentin formation from the odontoblast-like cells and partially from fibroblasts. Considering that tooth maturation and bacteria invasion can cause intratubular and peritubular ex novo dentin formation, dentinal surface does not have to be considered constant in time [32, 34, 35].

Our research has been conducted on premolars in paediatric-aged patients, extracted for orthodontic reasons, in order not to include excessive variables in the research data.

The results of our research showed that dentinal structure varied in the different root canal portions. This anatomic peculiarity can explain the differences identified in the adhesive efficiency in the different endocanalar regions.

The dentinal microscopic structure is an important topic in conservative dentistry for the choice of different adhesive technologies and for a correct clinical approach.

## Figures and Tables

**Table 1:** Average and St. Dev. of tubular number and tubular diameter (three observations/areas of 400 *µ*m^2^).

	Sample 1	Sample 2	Sample 3	Sample 4	Sample 5	Sample 6	Sample 7	Sample 8	Sample 9	Sample 10
Portion	Tubular number									
Apical	17	6	21	23	11	13	19.33	23	25	22
Middle	12	13.66	12.66	11.33	11	7.66	16.6	14	18	7.12
Coronal	14.66	17.33	23.33	12	14.33	15	23	21	26	22

Portion	Tubular diameter									
Apical	0.69	3.36	0.62	0.56	2.21	1.93	2.17	2.34	2.28	2.1
Middle	6.11	2.32	2.72	3.12	2.6	4.69	6.64	6.35	5.97	6.03
Coronal	3.36	3.08	3.38	5.38	4.05	5.8	5.9	5.05	3.69	6.43

	Sample 11	Sample 12	Sample 13	Sample 14	Sample 15	Sample 16	Sample 17	Sample 18	Sample 19	Sample 20

Portion	Tubular number									
Apical	15	20	18	14	22	24	14.33	22	17	14
Middle	12.33	5.33	15.66	8.66	15.33	12	14.66	16.33	15.33	13.33
Coronal	20.66	12.33	16.33	13	15	13.66	24	23.66	18.33	23

Portion	Tubular diameter									
Apical	0.67	0.58	2.11	3.2	2.15	2.33	2.56	2.54	0.54	0.66
Middle	1.7	3.13	3.08	3.17	2.41	2.64	2.36	2.98	3.48	3.27
Coronal	2.44	4.38	4.2	3.97	2.57	3.57	4.25	5.7	3.33	5.76

	Sample 21	Sample 22	Sample 23	Sample 24	Sample 25	Sample 26	Sample 27	Sample 28	Sample 29	Sample 30

Portion	Tubular number									
Apical	21	20.33	14	15	23	22	17	16	14.66	18.66
Middle	13.66	11.66	12.66	11.33	7.33	15	14.66	7.33	11.33	13.33
Coronal	21.33	18.33	14.66	19.66	15.33	24	24.66	16.33	11.33	23.33

Portion	Tubular diameter									
Apical	2.26	2.73	0.55	2.44	2.23	2.72	0.41	1.97	0.46	0.56
Middle	2.42	2.86	4.79	3.21	6.23	2.46	3.47	6.12	2.7	3.44
Coronal	5.27	3.9	3.54	4.2	4.67	5.37	4.21	4.1	3.76	4.41

		Tubular number				Tubular diameter				
		Portion	Average	St. Dev.		Portion	Average	St. Dev.		

		Apical	18.077	4.35		Apical	1.731	0.93		
		Middle	12.376	3.10		Middle	3.749	1.48		
		Coronal	18.586	4.25		Coronal	4.324	1.00		

**Table 2:** Observation data (mm^2^ and %) for each root portion.

Portion	Tubular number (mm^2^)	Tubular lumen area (*μ*m^2^)	Surface occupied by tubules (1 mm^2^)	Intertubular area (mm^2^)	Tubular lumen area/dentinal surface (%)
Apical	45,192 ± 10,888	3.033 ± 2.43	0.14	0.86	13.71%
Middle	30,940 ± 7,651	12.77 ± 10.23	0.40	0.60	39.53%
Coronal	46,798 ± 10,644	15.47 ± 7.06	0.72	0.28	72.42%
